# Parent-Reported Feeding Difficulties and Speech and Language Development in Late Preterm Infants

**DOI:** 10.3390/children13020204

**Published:** 2026-01-31

**Authors:** Nina Žumer, Irena Hočevar Boltežar, Lilijana Kornhauser Cerar

**Affiliations:** 1 Department of Speech and Language Therapy, Faculty of Education, University of Ljubljana, 1000 Ljubljana, Slovenia; 2 Department of Otorhinolaryngology, Faculty of Medicine, University of Ljubljana, 1000 Ljubljana, Slovenia; irena.hocevar-boltezar@mf.uni-lj.si; 3 Division of Gynaecology and Obstetrics, Department of Perinatology, University Medical Center Ljubljana, 1000 Ljubljana, Slovenia

**Keywords:** late preterm infants, feeding difficulties, speech and language disorders, feeding support

## Abstract

*Background/Objectives:* This study aimed to compare feeding difficulties (FDs) in Slovenian late preterm infants (LPIs) and full-term infants (FTIs), to identify factors associated with FDs, and to examine a possible association between FDs and later speech–language disorders (SLDs) among LPIs. *Methods:* Parents of 177 children (n_LPI_ = 89; n_FTI_ = 88) born at Ljubljana Maternity Hospital between 1 July 2021 and 30 June 2022 completed a questionnaire providing general information on their child, along with the child’s health, birth history, and development of feeding, swallowing, and speech-language skills. Additional clinical data were obtained from medical documentation. Using these data, comparisons were made between LPIs and FTIs and within the LPI group according to the presence or absence of FDs and SLDs. *Results:* LPIs experienced significantly more FDs than FTIs (32.6% vs. 11.4%, *p* < 0.001). They were breastfed less frequently (68.5% vs. 94.3%, *p* < 0.001) and had a shorter breastfeeding duration (13.8 months vs. 17.3 months, *p* = 0.038). LPIs also demonstrated more challenges in speech and language development (34.1% vs. 15.3%, *p* = 0.004). However, no significant association was found between FDs and later SLDs (*p* = 0.957). *Conclusions:* LPIs are at increased risk of both FDs and SLDs compared with FTIs. These findings highlight the importance of high-quality preventive care and timely multidisciplinary interventions.

## 1. Introduction

Late preterm infants (LPIs), born between 34^+0^ and 36^+6^ weeks of gestation [1], account for 65–75% of all preterm births in high-income countries [2]. Their proportion has been increasing in recent decades, highlighting their growing public health significance [3,4]. Although physiologically more mature than very preterm infants, LPIs remain at elevated risk of neonatal morbidity and mortality [5]. Compared with full-term infants (FTIs), LPIs are more susceptible to respiratory complications [6], thermoregulation problems [7,8], and feeding difficulties (FDs), which can contribute to or coexist with other common complications such as hypoglycaemia, dehydration, and hyperbilirubinaemia [7,8,9,10]. Their relatively immature immune system predisposes them to higher rates of infection [9,10]. Consequently, LPIs more often require intensive care [6] and experience longer hospitalisations [10], with increased rates of emergency department visits and rehospitalisations after discharge—most commonly due to jaundice, but also because of FDs, respiratory problems, and fever [11].

FDs in LPIs primarily arise from the developmental processes of sucking and swallowing. The first signs of non-nutritive sucking and pharyngeal swallowing appear around 15 weeks of gestation, with basic sucking and swallowing skills developed by week 28 [12,13,14]. However, effective oral feeding requires precise coordination of sucking, swallowing, and breathing, which matures only after birth. In premature infants, this coordination typically develops around 37 weeks postmenstrual age. Between 34^+0^ and 36^+6^ weeks postmenstrual age, this coordination is often incomplete, leading to substantial variability in feeding abilities among LPIs [15,16,17].

FDs affect 40–60% of LPIs, with incidence inversely related to gestational age (GA) [8]. They are most often characterised by difficulties initiating and maintaining breastfeeding, delayed oral feeding, and fatigue during feeding [18]. Although breastfeeding is well recognised for its health benefits [19], LPIs are rarely exclusively breastfed [20]. Contributing factors include poor sucking-swallowing-breathing coordination, lower sucking pressure, lower general muscle tone, disturbed sleep–wake cycles, general fatigue, early maternal-infant separation [18], and various pregnancy-related conditions that predispose to preterm birth or delay lactogenesis, such as multiple pregnancy, gestational diabetes, or caesarean section [21]. Consequently, LPIs and their mothers often require additional professional support with feeding. Even though early support in clinical settings is crucial for successful breastfeeding establishment [22,23,24], it is often not consistently provided [25]. Nutritional management strategies also vary, as evidence-based guidelines for LPIs remain limited [26]. FDs may persist into toddlerhood: while the introduction of solid food is generally unaffected [27,28], LPIs are more prone to oral-motor issues and picky eating at the age of two [29].

LPIs are also at increased risk of speech and language disorders (SLDs) [30,31,32,33,34,35], although findings are inconsistent [36]. Evidence suggests that these risks may diminish with age [35], and by primary school, no significant differences between LPIs and FTIs are observed [37]. As feeding and speech use common anatomical structures (oral cavity, pharynx, larynx), early feeding development may influence later articulation skills; however, the existing literature on this association is sparse and methodologically limited [38,39,40].

Despite their clinical and public health importance, no study in Slovenia has yet addressed FDs in LPIs. Therefore, the present study aimed to (1) assess the risk of FDs in LPIs compared with FTIs, (2) describe the nature of these difficulties in LPIs, (3) identify factors associated with FDs, and (4) explore possible associations between FDs and later SLDs among LPIs. The findings may help improve medical support for LPIs and their families.

## 2. Materials and Methods

### 2.1. Participants

**Figure 1:**
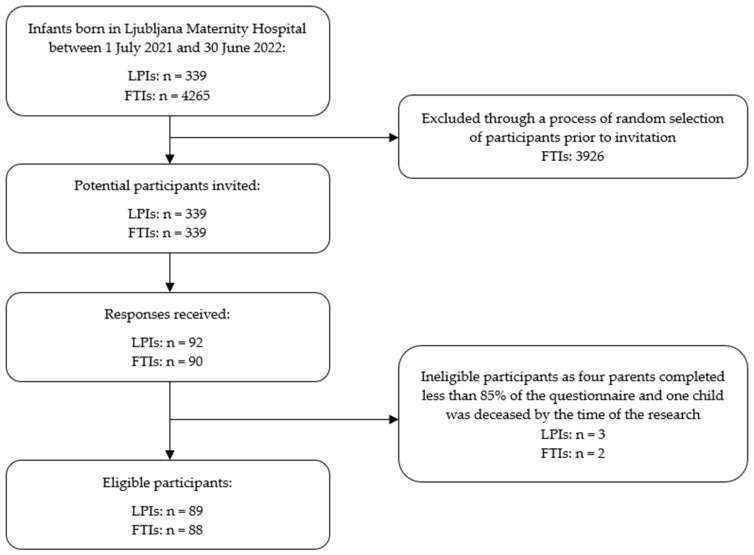
Flowchart of participant recruitment process.

The parents of all 339 LPIs born within a one-year period were invited to participate in this study, and an equal number of FTIs were randomly selected as a comparison group. The inclusion criteria for the children were birth at Ljubljana Maternity Hospital between 1 July 2021 and 30 June 2022 and survival until the time of data collection. Children whose parents completed less than 85% of the questionnaire were excluded from the study. A total of 177 children were included in the study (n_LPI_ = 89, n_FTI_ = 88), with their mothers acting as surrogates, providing all information and informed consent for their child’s participation (Figure 1). In addition, 106 mothers also provided consent for the research team to review their child’s medical documentation for the purpose of this study.

### 2.2. Sample Size Calculation

Two a priori sample size calculations were conducted for the two main study aims.

First, to compare the prevalence of FDs between LPIs and FTIs, prevalence estimates reported in the literature were used. Given the wide variability in reported rates of FDs (20–50% in FTIs and 35–60% in LPIs), prevalence estimates of 35% for FTIs and 45% for LPIs were assumed. A χ^2^ test with a two-sided significance level of α = 0.05 and statistical power of 0.80 was applied, resulting in a minimum required sample size of 82 participants per group. The final sample included 89 LPIs and 88 FTIs, meeting the calculated requirements and providing sufficient power to detect the assumed group difference.

Second, to examine the association between FDs and SLDs among LPIs, an a priori power analysis for the χ^2^ test was performed, assuming a medium effect size (w = 0.30), statistical power of 0.80, and a significance level of α = 0.05. The required sample size was 88 participants. The final sample comprised 89 LPIs, thereby meeting the calculated sample size requirement and providing adequate statistical power to detect a medium association between FDs and SLDs.

### 2.3. Instruments and Measures

Data were collected using a structured online parental questionnaire designed specifically for this study. The questionnaire was adapted from the Feeding and Swallowing Disorders in Preterm Infants unvalidated questionnaire developed by Slana et al. [41], which has already been used in the clinical environment of Ljubljana Maternity Hospital. It covered four domains:

1. General child data: sex, date of birth, birth order.
2. Birth and early health history: pregnancy complications, delivery details, GA at birth, birth weight and length, neonatal health status, hospitalisation details, need for special care.
3. Feeding and swallowing development: parental feeding intentions before birth, timing of first breastfeeding, breastfeeding duration, breastfeeding difficulties, and other information related to infant feeding before and after discharge from the maternity hospital.
4. Speech and language development: age at first word and first sentence, presence of SLD, and need for speech and language therapy.

The questionnaire also included a statement asking parents for permission to review the child’s medical documentation for the purposes of this study. Medical records containing information on the infants’ birth weight, birth length, perinatal complications, and length of hospitalisation were used primarily to verify and, where necessary, supplement parental reports, thereby minimising the potential impact of recall bias. If parents did not provide consent to access their child’s medical documentation, analyses relied solely on questionnaire data.

#### Definitions

In the present study, feeding difficulties (FDs) were defined primarily as problems with breast- or bottle feeding during infancy, not with the intake of solid foods (although some participants may have experienced difficulties with both milk and solid feeding). FDs were identified solely based on parental reports and were considered present if all three of the following criteria were met:

1. Feeding problems during the first days after birth;
2. Specific challenges with sucking at the breast or bottle (e.g., latching difficulties, poor sucking strength, fatigue during feeding, clicking sounds, suck-swallow-breathe incoordination) before and after discharge from the maternity hospital;
3. At least one sign of a feeding and swallowing disorder (e.g., choking, vomiting, feeding refusal, excessive air swallowing, difficulties managing saliva, feeding lasting more than 30 min, abnormal oral sensitivity, insufficient food intake) before and after discharge from the maternity hospital.

The term speech and language disorders (SLDs) was used to refer to speech and language developmental delays, including developmental language disorders and speech-sound disorders.

### 2.4. Data Collection Procedures

This study was an analytical cross-sectional study with a control group. The parents of 678 children were invited to participate in this study in September 2024. Their addresses were obtained from maternity hospital records. Invitation letters, including a link and a QR code to the online questionnaire, were sent to them. Parental questionnaires were collected in November 2024. Feeding difficulties and speech-language outcomes were assessed in all participants using the same questionnaire. Subsequent stratification within the LPI group according to the presence or absence of FDs and SLDs was performed for analytical purposes to explore factors associated with FDs and SLDs.

### 2.5. Data Analysis

Statistical analyses were conducted using IBM SPSS Statistics 30.0. Descriptive statistics (frequencies, percentages, arithmetic means, standard deviations, minimum and maximum values) were calculated for all participants and separately for LPIs and FTIs. Comparisons between the groups were performed using χ^2^ tests (with Likelihood Ratio correction where appropriate), independent samples *t*-tests (with Welch’s correction for unequal variances when necessary), Mann–Whitney U tests, and McNemar tests. Normality and homogeneity were assessed using Kolmogorov–Smirnov and Levene’s tests. Using the same procedures, subgroups of LPIs with and without FDs, and LPIs with and without SLDs were compared. Multivariable logistic regression was conducted to identify factors associated with FDs and SLDs in LPIs. Statistical significance was determined at *p* < 0.05.

### 2.6. Ethics

This study was conducted in accordance with the Code of Ethics of the World Medical Association (Declaration of Helsinki) and was approved by the National Medical Ethics Committee of the Republic of Slovenia (Protocol No. 0120-407/2024-2711-3, dated 16 October 2024).

By completing the questionnaire, all participants provided informed consent. Participation was voluntary and anonymous; personal data were coded and stored in accordance with legislative requirements. For parents who provided explicit informed consent, their child’s hospital records were retrieved by the research team and linked to the questionnaire data using a study ID number.

## 3. Results

A total of 177 mothers completed the questionnaire: 89 had LPIs and 88 had FTIs. At the time of this study, the children were aged 28–40 months. As shown in Table 1, the groups did not differ significantly in terms of gender distribution or age at the time of the study. They were also similar in terms of the prevalence of delivery complications and general health issues of the child. However, significant differences were found between LPIs and FTIs in all other birth-related variables, including birth weight and length, GA, mode of delivery, foetal plurality, hospitalisation variables, and pregnancy and neonatal health complications.

As presented in Table 1, the groups differed significantly in their feeding modalities. LPIs were less likely than FTIs to be exclusively or partially breastfed, despite mothers reporting similar intentions to breastfeed prior to delivery. Significantly more LPIs than FTIs experienced feeding difficulties in the postnatal ward and after discharge from the maternity hospital. They also exhibited a higher prevalence of specific challenges with breast- and bottle feeding, most commonly fatigue during feeding (44.9%), regurgitation or vomiting (39.3%), insufficient sucking strength (36.0%), and latching difficulties (32.6%). Only 5.6% of mothers reported difficulties with their child’s suck-swallow-breathe coordination. LPIs also remained in hospital longer after birth than FTIs (mean stay 7.09 ± 4.67 days vs. 3.09 ± 1.37 days). However, more than 90% of included LPIs were discharged from the maternity hospital within 16 days. This indicates that the great majority began to gain weight properly and the medical staff no longer observed any significant FD.

On the other hand, no significant differences were observed between LPIs and FTIs in terms of the prevalence of reported signs of feeding and swallowing disorders, such as choking, vomiting, feeding refusal, excessive air swallowing, difficulties with saliva management, prolonged feeding times (>30 min), abnormal oral sensitivity, and insufficient food intake. Maternal satisfaction with information on newborn feeding provided until discharge from the postnatal ward did not differ significantly between the groups. Additional analyses showed that while the child’s GA was not associated with maternal satisfaction, the child’s birth order and maternal satisfaction with information were significantly related (χ^2^ (4) = 30.009, *p* < 0.001). More than half of first-time mothers (57.6%) and only 19.6% of mothers with at least two children reported feeling inadequately informed about infant feeding upon discharge.

In accordance with this study’s definition, FDs were identified in 29 LPIs (32.6%) and 10 FTIs (11.4%), with a significant difference between the two groups (*p* < 0.001). Further analysis within the LPIs group revealed that the FD prevalence was higher in subgroups with lower GA at birth (41.7% vs. 34.5% vs. 25.0% for 34, 35, and 36 weeks GA, respectively).

To explore factors associated with FDs among LPIs, comparisons were made between LPIs with and without FDs (as shown in Table 2). Only variables showing a statistically significant association and a plausible clinical relevance were considered for further logistic regression analysis. Breastfeeding in the first hour after birth was the only variable showing a significant association with FDs in bivariate analysis (Table 2); however, in the multivariable logistic regression model, this association did not remain statistically significant (OR = 0.95, 95% CI [−0.120, 0.010], *p* = 0.094). Thus, no variables were identified as being independently associated with FDs among LPIs in the logistic regression analysis.

As also presented in Table 1, LPIs experienced more frequent delays in speech and language development compared to FTIs. On average, they produced their first words and first sentences later, had a higher prevalence of SLDs, and were more often referred to a speech-language pathologist. Not all parents provided complete information about their child’s speech and language development; therefore, the statistics are based on the available data, and differences in sample size are indicated in Table 1.

To identify factors associated with SLDs among LPIs, bivariate (Table 3) and logistic regression analyses were conducted. Variables showing a statistically significant association with SLDs in bivariate analysis and deemed clinically relevant were included in the logistic regression model. Speech and language development variables were not included, as they represent manifestations or consequences rather than explanatory factors for SLDs. Logistic regression identified the child’s general health issues as independently associated with SLDs (OR = 4.34, 95% CI [1.240, 15.182], *p* = 0.022). LPIs with developmental or congenital health conditions (e.g., hypo- or hypertonia, gastro-oesophageal reflux, congenital heart conditions, atopic dermatitis, or other) had significantly higher odds of developing an SLD compared with LPIs without such conditions. No other variables remained significantly associated with SLDs in the multivariate analysis.

Although LPIs showed a higher prevalence for both FDs and SLDs, no significant association was observed between the two variables (*p* = 0.957), suggesting that early FDs were not associated with later SLDs in LPIs.

## 4. Discussion

Although several attempts have been made to define paediatric FDs [42,43], no universally accepted definition or standardised inclusion criteria currently exist. This lack of conceptual clarity, combined with differences between clinical and parental perspectives on FDs, complicates comparisons across studies. There have been few studies on FDs in LPIs. The reported prevalence of FDs among LPIs ranges from 32% to 60% [8,44,45,46], depending strongly on how FDs are defined. In our study, 37.1% of parents reported FDs during the postnatal ward stay, whereas 68.5% identified at least one specific challenge with sucking at the breast or bottle.

Several factors may explain this discrepancy. In the intensive care unit and the postnatal ward, there are nurses and physiotherapists specialised in lactation counselling. On discharge from the hospital, mothers receive oral information on newborn care, and a community public health nurse is notified to visit the newborn as soon as possible. FDs may emerge or become more apparent after discharge, when lactation is fully established [45] or when parents become solely responsible for infant care [47]. FDs may also develop later due to interactional challenges within the parent–child relationship [48], changes in feeding practices [49], or subsequent health conditions such as food sensitivities, reflux, or colic [48]. The methodological characteristics of our questionnaire could also have contributed to the observed difference: parents were first asked whether their child had any FDs, followed by a list of specific signs. Thus, some parents may have recognised particular features of their child’s feeding behaviour only when prompted, even if they did not initially perceive their child as having FDs.

In addition to biological and methodological factors, insufficient and inconsistent feeding support provided to parents is likely to play an important role in the occurrence or delayed recognition of FDs. Almost 40% of mothers participating in our study reported feeling inadequately informed about infant feeding at the time of discharge from the postnatal ward. Among primiparas, 57.6% felt inadequately informed, whereas only 19.6% of multiparas reported feeling this way. As we did not have detailed information on the mothers’ participation in antenatal classes on newborn care, educational background, or family socioeconomic status, we were unable to examine potential associations between these factors and feelings of being adequately informed about infant feeding. Nevertheless, regardless of the underlying reasons, this finding is concerning and calls for an evaluation of health education programmes for expectant and new mothers, as well as institutions providing postnatal support. Feeling inadequately prepared places considerable stress on new mothers, potentially exacerbating FDs [18,50].

Consistent with those of previous research [7,8,9,10], our findings confirm that LPIs are at higher risk for FDs than FTIs. In our sample, 32.6% of LPIs were classified as having FDs—that is, FDs on the postnatal ward or after discharge from the maternity hospital, at least one specific challenge with sucking at the breast or bottle, and at least one sign of feeding and swallowing disorders. Earlier studies attributed this increased vulnerability primarily to immature sucking skills and inadequate suck-swallow-breathe coordination, which are still developing between 34^+0^ and 36^+6^ weeks postmenstrual age [15,16,17]. Interestingly, only 5.6% of parents in our study reported difficulties in suck-swallow-breathe coordination among their LPIs. This does not necessarily indicate a lower prevalence of such issues in our cohort but rather suggests that parents are more likely to recognise and report overt symptoms (e.g., regurgitation, prolonged feeding times, latching difficulties) than subtle physiological mechanisms such as poor suck-swallow-breathe coordination. These findings highlight the importance of combining parental reports with clinical assessments in future studies to more accurately characterise the nature and underlying mechanisms of FDs [51,52].

Breastfeeding is one of the most clinically relevant domains in which FDs manifest among LPIs [18]. This was clearly reflected in our findings, which showed that exclusive breastfeeding rates were significantly lower among LPIs compared to FTIs (48.3% vs. 86.4%), with a shorter overall breastfeeding duration (14 vs. 17 months), despite similar maternal intentions to breastfeed prior to delivery. These trends have already been consistently documented in other studies [53,54,55] and are mainly attributed to the combined influence of LPIs’ biological immaturity, weak sucking strength, and perceived insufficient milk supply in the early postnatal period and the early introduction of solid foods before four months of age [55]. These difficulties are further associated with increased maternal psychological distress, which is more common among mothers of LPIs [18,55]. Importantly, previous research has demonstrated that with adequate and timely professional support, LPIs can substantially improve their breastfeeding performance in the first months of life [55,56]. This shows that breastfeeding is not only a marker of feeding ability but also an outcome that can be substantially modified through structured interventions, underscoring the critical importance of early and continuous lactation support for this population. Clinical practice in postnatal wards—particularly the availability and quality of lactation support—plays a decisive role in establishing and maintaining breastfeeding, either facilitating or hindering it [22,23,24,53]. Therefore, systematic integration of early lactation support into routine postnatal care for LPIs is essential. Integrating speech-language pathologists into Slovenian NICU teams and postnatal wards could further enhance the quality of lactation support in the early days after birth.

While numerous studies have consistently shown that LPIs are at increased risk of FDs, the predictive factors underlying this risk remain less well understood. In our study, no factors associated with FDs were identified. Previous research has emphasised GA as one of the strongest predictors of FDs, with lower GA at birth or postmenstrual age at the time of oral feeding initiation associated with a higher likelihood of FDs [8,17,57,58]. Although this trend was also observed in our cohort, with FD prevalence decreasing as GA increased, the association was not statistically significant. Interestingly, early initiation of breastfeeding (within the first hour after birth) was significantly associated with FDs in univariate analysis but did not remain significant in the multivariable logistic regression. This finding contrasts with those of previous studies [59,60,61] that primarily examined breastfeeding success, whereas our outcome focused on overall feeding performance. These differences in study endpoints likely explain the discrepancy.

Beyond GA, other studies have identified several factors associated with FDs among preterm infants, including neonatal morbidities [17], hypotonia and lower socio-economic status [62], early maternal-infant separation [18,50], and clinical indicators such as low birth weight, a low Apgar score, or prolonged mechanical ventilation [57,58]. However, these factors have been investigated mainly in the broader preterm population. Factors associated with FDs specifically in LPIs remain insufficiently explored, highlighting the need for future research focusing specifically on this subgroup.

In our study, there were significantly more children with FDs after discharge from the hospital among LPIs than among FTIs (23.6% vs. 10.2%, respectively). Therefore, it is very important that they have professional help when they are in home surroundings. Infants are discharged from the maternity hospital only after they have gained sufficient weight and show no significant feeding problems. In Slovenia, mothers and infants receive special postnatal care provided by a community public health nurse, who offers structured home visits after discharge from the maternity hospital. This follow-up care typically lasts for several months, with extended support in certain regions. Infants with FDs can receive additional care if needed.

Our findings are consistent with those of previous studies showing a higher prevalence of SLDs among LPIs compared to FTIs [30,31,32,33,34,35]. Although associations between FDs and SLDs have been reported in broader preterm populations [38,39,63,64], and a longer breastfeeding duration even acts as a protective factor against later SLDs [65], our study did not confirm an association between FDs and SLDs specifically in LPIs. The absence of statistically significant findings does not necessarily indicate the absence of a true effect, as weaker associations may have remained undetected due to the methodological features of our study. It may also reflect the limited accuracy of parental reports on speech and language development. As the data on our cohort are therefore limited, the topic should be further explored; however, future studies should integrate subjective parental reports on children’s speech and language development with objective clinical assessments by speech-language pathologists [66].

Developmental or congenital health conditions (e.g., hypotonia, hypertonia, gastro-oesophageal reflux, atopic dermatitis, heart conditions) were found to be significantly associated with SLDs among LPIs. Interestingly, maternal intention to breastfeed was also associated with SLDs, whereas actual feeding methods were not. Mothers of LPIs with SLDs were less likely to intend to breastfeed. Although this association may seem illogical, similar findings have been reported previously [67], suggesting that mothers who intend to breastfeed are more likely to seek diverse sources of information on infant feeding and to demonstrate higher levels of knowledge regarding child development and health awareness. The higher prevalence of developmental or congenital health conditions in our LPI-SLD subgroup may partially reflect this pattern. Further research is required to clarify the links between child health, feeding intention, and speech-language development in LPIs. Nevertheless, providing parents of these children with clear information about early communication milestones and practical strategies to support communication development in the home environment may be beneficial. Importantly, support for parent–child interactions should be offered from the first days after birth and provided directly to the LPIs, rather than waiting until they reach full term or later developmental stages [68]. The inclusion of a trained speech-language pathologist in the postnatal ward team would facilitate the provision of such guidance. In later follow-ups, paediatricians should closely monitor speech and language development in LPIs with underlying health conditions and ensure timely referral to a speech-language pathologist when needed.

Overall, these findings highlight the need for standardised definitions and assessment tools for feeding difficulties, which could enable more consistent research and clinical practice across settings. Early identification and targeted support for LPIs experiencing FDs could improve breastfeeding outcomes and potentially mitigate downstream risks such as SLDs. Integrating structured parental questionnaires with clinical feeding assessments by institutions for postnatal support may enhance early detection of FDs. Moreover, given the observed link between congenital or developmental health conditions and SLDs, paediatric follow-up programmes for these children should include routine speech and language screening and provide families with early intervention resources. At the public health level, these results emphasise the importance of strengthening postnatal care and breastfeeding support programmes for LPIs—including early lactation support, interdisciplinary collaboration, and accessible speech-language services—to support overall infant health and optimise long-term developmental outcomes.

There are several limitations to our study. The first limitation is a relatively small sample size. Although the final sample of 89 LPIs and 88 FTIs was sufficient for group comparisons and for detecting a medium association between FDs and SLDs among LPIs, it may have lacked sufficient power to identify smaller or more subtle associations. Therefore, effect size estimates should be interpreted with caution.

Second, the researchers did not have access to medical records of the entire study population (339 LPIs and 339 FTIs, N = 678) due to ethical and consent limitations. As a result, it was not possible to assess whether the recruited sample was fully representative of the total population, which may have introduced some selection bias.

Third, due to the considerable variability in reported prevalence rates of FDs and the lack of reliable prevalence data for SLDs in LPIs, the sample size calculation was based on an assumed effect size rather than precise epidemiological estimates. While this approach is methodologically acceptable, it may limit the generalisability of the findings. Future studies using larger, population-based samples are needed to further explore the relationship between FDs and SLDs in LPIs.

Fourth, the sample was clinically heterogeneous, including LPIs and FTIs with congenital anomalies. In practice, these children were few in number, so their impact on the overall results is likely limited; nevertheless, this heterogeneity may have introduced some variability in feeding and speech-language outcomes.

Regarding the age of the children, we wanted to include children whose speech-language development had already begun, while ensuring that their birth was not too distant in time so that parents could recall their child’s feeding difficulties during their first months of life. The selected age range (28–40 months) allowed for assessment of the emergence of the first word and sentences in the participating children. However, some children have delayed speech development due to other reasons, such as genetic predisposition or hearing impairment, for which we did not have available data. Similarly, information on the parental education level and family socioeconomic status—both of which may influence speech-language development—was not collected. The absence of data on families’ socioeconomic status and parental education is also a limitation regarding the reported occurrence of FDs by parents.

Finally, an important limitation of this study was the use of a non-validated questionnaire and the subjective nature of parental responses to the questionnaire. The inclusion of objective assessments or expert evaluations of FDs and SLDs would likely enhance the accuracy of our findings.

## 5. Conclusions

This study demonstrated that LPIs are at increased risk for both FDs and SLDs. No variables were found to be independently associated with FDs, whereas general health conditions were significantly associated with SLDs. Furthermore, no significant association was found between FDs and SLDs among LPIs.

Despite all the mentioned limitations, the findings have important clinical implications for LPIs and their families. They highlight the need for comprehensive multidisciplinary support for LPIs and their families, particularly in establishing and maintaining effective oral feeding, promoting breastfeeding, and ensuring timely prevention and intervention with regard to speech and language disorders.

## Figures and Tables

**Table 1:** Comparison of general and birth variables, feeding variables, and speech and language variables between late preterm infants (LPIs) and full-term infants (FTIs).

Variable		LPIs (n = 89)	FTIs (n = 88)	*p*
**General and birth variables**				
Male gender, *n* (%)		43 (48.3%)	49 (55.7%)	0.327
Age at time of study, mos, M ± SD		34.28 ± 3.67	34.55 ± 3.56	0.701
Gestational age, wks, M ± SD		35.13 ± 0.82	39.85 ± 0.74	
Birth weight, g, M ± SD		2471.02 ± 543.61	3582.50 ± 384.94	<0.001
Birth length, cm, M ± SD		46.81 ± 3.02	52.00 ± 1.79	<0.001
Multiple pregnancy, *n* (%)		24 (27.0%)	0 (0%)	<0.001
Caesarean delivery, *n* (%)		32 (36.0%)	13 (14.8%)	<0.001
Hospitalisation length, days, M ± SD		7.09 ± 4.67	3.09 ± 1.37	<0.001
Maternal-infant separation, *n* (%)		31 (34.8%)	11 (12.5%)	<0.001
Separation length, days, M ± SD		4.63 ± 4.58	2.59 ± 3.97	<0.001
Pregnancy complications, *n* (%)		60 (67.4%)	31 (35.2%)	<0.001
Delivery complications, *n* (%)		8 (9.0%)	11 (12.5%)	0.329
Neonatal complications, *n* (%)		58 (65.2%)	19 (21.6%)	<0.001
Child’s general health issues, *n* (%)		29 (32.6%)	18 (20.5%)	0.068
**Feeding variables**				
Feeding method during the first six months of life	Exclusive breastfeeding, *n* (%)	43 (48.3%)	76 (86.4%)	<0.001
	Bottle feeding, *n* (%)	28 (31.4%)	5 (5.7%)	
	Mixed feeding, *n* (%)	18 (20.3%)	7 (7.9%)	
Exclusive breastfeeding intentions, *n* (%)		81 (91.0%)	82 (93.2%)	0.122
Breastfeeding duration, mos, M ± SD		13.82 ± 10.06	17.32 ± 8.83	0.038
Breastfeeding in first hour after birth, *n* (%)		39 (43.8%)	68 (77.3%)	<0.001
Feeding problems in postnatal ward, *n* (%)		33 (37.1%)	15 (17.0%)	0.003
Feeding problems after discharge from maternity hospital, *n* (%)		21 (23.6%)	9 (10.2%)	0.018
Breast- or bottle feeding challenges, *n* (%)		61 (68.5%)	30 (34.1%)	<0.001
Signs of feeding and swallowing disorders, *n* (%)		53 (59.6%)	47 (53.4%)	0.410
Adequately informed about newborn feeding, *n* (%)		53 (59.6%)	58 (64.8%)	0.474
**Speech-language variables**				
Age at first word, mos, M ± SD		13.65 ± 4.44	12.37 ± 3.72	0.045
		n = 86	n = 83	
Age at first sentence, mos, M ± SD		19.28 ± 5.06	17.22 ± 4.20	0.005
		n = 85	n = 81	
Speech and language disorders, *n* (%)		30 (34.1%)	13 (15.3%)	0.004
		n = 88	n = 85	
Referral to SLP, *n* (%)		7 (7.8%)	4 (4.8%)	0.048
		n = 89	n = 84	

Note: M = mean, SD = standard deviation, *n* = numerus, n = number of participants who provided information for this variable, g = grams, cm = centimetres, wks = weeks, mos = months, SLP = speech and language pathologist. All analyses are unadjusted. Bold text indicates category labels, not individual variables.

**Table 2:** Factors associated with feeding difficulties (FDs) among late preterm infants (LPIs): a comparison of LPIs with and without FDs.

Variable		LPIs With FDs (n = 29)	LPIs Without FDs (n = 60)	*p*
Male gender, *n* (%)		10 (34.5%)	33 (55.0%)	0.069
Gestational age, wks, M ± SD		34.97 ± 0.82	35.22 ± 0.80	0.174
Birth weight, g, M ± SD		2456.32 ± 419.35	2478.18 ± 604.40	0.843
Birth length, cm, M ± SD		46.71 ± 2.37	46.87 ± 3.31	0.817
Multiple pregnancy, *n* (%)		8 (27.6%)	16 (26.7%)	0.927
Caesarean delivery, *n* (%)		9 (31.0%)	23 (38.3%)	0.501
Maternal-infant separation, *n* (%)		11 (37.8%)	20 (33.3%)	0.670
Separation length, days, M ± SD		3.77 ± 3.78	5.10 ± 5.00	0.604
		n = 11	n = 20	
Pregnancy complications, *n* (%)		22 (75.9%)	38 (63.3%)	0.237
Delivery complications, *n* (%)		3 (10.3%)	5 (8.3%)	0.758
Neonatal complications, *n* (%)		22 (75.9%)	36 (60.0%)	0.141
Child’s general health issues, *n* (%)		12 (41.4%)	17 (28.3%)	0.218
Feeding method during the first six months of life	Exclusive breastfeeding, *n* (%)	12 (41.4%)	31 (51.7%)	0.682
	Bottle feeding, *n* (%)	9 (31.1%)	19 (31.6%)	
	Mixed feeding, *n* (%)	8 (27.5%)	10 (16.7%)	
Breastfeeding in first hour after birth, *n* (%)		18 (62.1%)	21 (35.0%)	0.035
Adequately informed about newborn feeding, *n* (%)		16 (55.2%)	37 (61.7%)	0.559

Note: M = mean, SD = standard deviation, *n* = numerus, n = number of participants who provided information for this variable, g = grams, cm = centimetres, wks = weeks. All analyses are unadjusted.

**Table 3:** Factors associated with speech-language disorders (SLDs) among late preterm infants (LPIs): a comparison of LPIs with and without SLDs.

Variable		LPIs With SLDs (n = 30)	LPIs Without SLDs (n = 58)	*p*
**General birth variables**				
Male gender, *n* (%)		13 (43.3%)	29 (50.0%)	0.553
Gestational age, wks, M ± SD		35.00 ± 0.83	35.21 ± 0.81	0.264
Birth weight, g, M ± SD		2300.17 ± 610.82	2547.69 ± 495.39	0.043
Birth length, cm, M ± SD		46.42 ± 2.95	46.95 ± 3.04	0.434
Multiple pregnancy, *n* (%)		12 (40.0%)	12 (20.7%)	0.054
Caesarean delivery, *n* (%)		16 (53.3%)	16 (27.6%)	0.017
Maternal-infant separation, *n* (%)		16 (53.3%)	15 (25.9%)	0.011
Separation length, days, M ± SD		6.03 ± 5.42	3.13 ± 2.98	0.075
		n = 16	n = 15	
Pregnancy complications, *n* (%)		21 (70.0%)	38 (65.5%)	0.672
Delivery complications, *n* (%)		3 (10%)	5 (8.6%)	0.832
Neonatal complications, *n* (%)		19 (63.3%)	38 (65.5%)	0.839
Child’s general health issues, *n* (%)		15 (50.0%)	14 (24.1%)	0.014
**Feeding variables**				
Feeding method during the first six months of life	Exclusive breastfeeding, *n* (%)	13 (43.3%)	30 (51.7%)	0.831
	Bottle feeding, *n* (%)	11 (36.7%)	17 (29.3%)	
	Mixed feeding, *n* (%)	6 (20.0%)	11 (19.0%)	
Exclusive breastfeeding intentions		83.3%	94.8%	<0.001
Breastfeeding duration, mos, M ± SD		8.93 ± 6.68	16.26 ± 10.48	0.016
		n = 15	n = 35	
Breastfeeding in first hour after birth, *n* (%)		10 (33.3%)	28 (48.3%)	0.016
Feeding problems in postnatal ward, *n* (%)		13 (43.3%)	20 (34.5%)	0.416
Breast- or bottle feeding challenges, *n* (%)		20 (66.7%)	40 (69.0%)	0.826
Signs of feeding and swallowing disorders, *n* (%)		18 (60.0%)	35 (60.3%)	0.975
Adequately informed about newborn feeding, *n* (%)		20 (66.7%)	33 (56.9%)	0.375
**Speech-language variables**				
Age at first word, mos, M ± SD		15.62 ± 4.82	12.52 ± 3.819	0.003
		n = 29	n = 56	
Age at first sentence, mos, M ± SD		21.83 ± 5.75	17.96 ± 4.13	0.002
		n = 29	n = 56	
Referral to SLP, *n* (%)		12 (40.0%)	4 (6.9%)	0.001
		n = 30	n = 56	

Note: M = mean, SD = standard deviation, *n* = numerus, n = number of participants who provided information for this variable, g = grams, cm = centimetres, wks = weeks, mos = months, SLP = speech and language pathologist. All analyses are unadjusted. Bold text indicates category labels, not individual variables.

## Data Availability

As the study is ongoing, institutional rules do not permit the release of raw data before its completion. The data will be available from the last author (LKC) upon reasonable request.

## Abbreviations

The following abbreviations are used in this manuscript:

LPI	Late preterm infant
FTI	Full-term infant
GA	Gestational age
FD	Feeding difficulty
SLD	Speech and language disorder
